# African swine fever virus protein MGF-505-7R promotes virulence and pathogenesis by inhibiting JAK1- and JAK2-mediated signaling

**DOI:** 10.1016/j.jbc.2021.101190

**Published:** 2021-09-10

**Authors:** Dan Li, Jing Zhang, Wenping Yang, Pan Li, Yi Ru, Weifang Kang, LuLu Li, Yong Ran, Haixue Zheng

**Affiliations:** 1 State Key Laboratory of Veterinary Etiological Biology and OIE/National Foot and Mouth Disease Reference Laboratory, Lanzhou Veterinary Research Institute, Chinese Academy of Agricultural Sciences, Lanzhou, Gansu, China; 2 State Key Laboratory of Virology, Wuhan Institute of Virology, Chinese Academy of Sciences, Wuhan, China

**Keywords:** ASFV, MGF-505-7R, RNF125, Hes5, JAK1, JAK2, ASFV, African swine fever virus, CAAS, Chinese Academy of Agricultural Sciences, FMDV, foot-and-mouth disease virus, HAD, hemadsorption, IRF1, IFN-regulatory factor 1, LVRI, Lanzhou Veterinary Research Institute, PAM, porcine alveolar macrophage

## Abstract

African swine fever virus (ASFV) is a large DNA virus that is highly contagious and pathogenic in domestic pigs with a mortality rate up to 100%. However, how ASFV suppresses JAK-STAT1 signaling to evade the immune response remains unclear. In this study, we found that the ASFV-encoded protein MGF-505-7R inhibited proinflammatory IFN-γ-mediated JAK-STAT1 signaling. Mechanistically, MGF-505-7R was found to interact with JAK1 and JAK2 and mediate their degradation. Further study indicated that MGF-505-7R promoted degradation of JAK1 and JAK2 by upregulating the E3 ubiquitin ligase RNF125 expression and inhibiting expression of Hes5, respectively. Consistently, MGF-505-7R-deficient ASFV induced high levels of IRF1 expression and displayed compromised replication both in primary porcine alveolar macrophages and pigs compared with wild-type ASFV. Furthermore, MGF-505-7R deficiency attenuated the virulence of the ASFV and pathogenesis of ASF in pigs. These findings suggest that the JAK-STAT1 axis mediates the innate immune response to the ASFV and that MGF-505-7R plays a critical role in the virulence of the ASFV and pathogenesis of ASF by antagonizing this axis. Thus, we conclude that deletion of MGF-505-7R may serve as a strategy to develop attenuated vaccines against the ASFV.

African swine fever (ASF) is a devastating infectious disease in swine with a mortality rate approaching 100% (1). The causative agent, ASF virus (ASFV), infects macrophages and antagonizes host innate immune responses to enhance its pathogenicity. Currently, no effective vaccine is available to prevent ASF, resulting in the continuous spread of the virus in Africa, Europe, and Asia. The first ASF outbreak in China in 2018 and subsequently more than 160 outbreaks have been declared so far (2, 3, 4, 5). Unfortunately, ASF continues to spread across China, causing devastating consequences to the development of the pig industry and domestic food security (6). Because the protective immunity and pathogenicity of ASFV are largely unknown, it is fundamentally important for us to explore the underlying mechanisms for the vaccine development and disease control.

ASFV, the only member of the *Asfarviridae* family, is a linear, nonsegmented, double-stranded DNA virus with a genome length ranging from 170 to 193 kilo base pairs, which encodes about 151 to 167 open reading frames (7, 8, 9, 10). It was previously reported that ASFV utilizes multiple self-encoding proteins to evade the host's innate and adaptive immune responses (11, 12, 13). For instance, MGF360 and MGF505/530 are believed to inhibit the induction of type I IFNs and their downstream antiviral interferon stimulated genes (14, 15). DP96R of ASFV targets both TBK1 and IKKβ to negatively regulate induction of antiviral cytokines (16). Additionally, ASFV Armenia/07 inhibits the cGAS-STING pathway by impairing STING activation during infection (17). Recently, we found that ASFV MGF-505-7R interacted with STING and degraded it by promoting the expression of the autophagy-related protein ULK1 (18). As most studies have focused on how ASFV antagonizes type I IFN responses, the underlying molecular mechanisms of ASFV impeding cellular type II interferon signaling pathway are currently unknown.

IFN-γ is a critical cytokine for the innate and adaptive immunity against viral and intracellular bacterial infections (19). The canonical IFN-γ-triggered signaling pathway is characterized by JAK-mediated phosphorylation of STAT1 (20). Previous studies have demonstrated that IFN-γ signaling pathways are activated through the binding of IFN-γ homo-dimer to IFNGR1 and IFNGR2, which results in spatial proximity of JAK1 and JAK2, leading to phosphorylation of IFNGR1 and JAKs (21, 22). The activation of both JAK1 and JAK2 cooperatively leads to the STAT1 phosphorylation, which induces the transcription of various genes involved in immune responses and cell proliferation (23, 24). The CXCLs are secreted from various cells by the stimulation of IFN-γ and act as chemotactic attractants of immune cells with a specific receptor CXCR3 (25, 26, 27).

In this study, we identified ASFV MGF-505-7R as a potent inhibitor of JAK-STAT1 signaling. ASFV MGF-505-7R interacted with JAK1 and JAK2 proteins and degraded them by upregulating RNF125 expression or downregulating Hes5 expression, respectively. Infection of pigs with MGF-505-7R-deficient ASFV showed an increased serum CXCL9 level, viral replication, and attenuated virulence and pathogenesis. Our findings suggest that the JAK-STAT1 axis mediates ASFV innate immunity, whereas evasion of JAK-STAT1 signaling by MGF-505-7R contributes to the virulence of the ASFV and pathogenesis of the ASF.

## Results

### ASFV impairs IFN-γ-induced transcription of downstream genes

**Figure 1:**
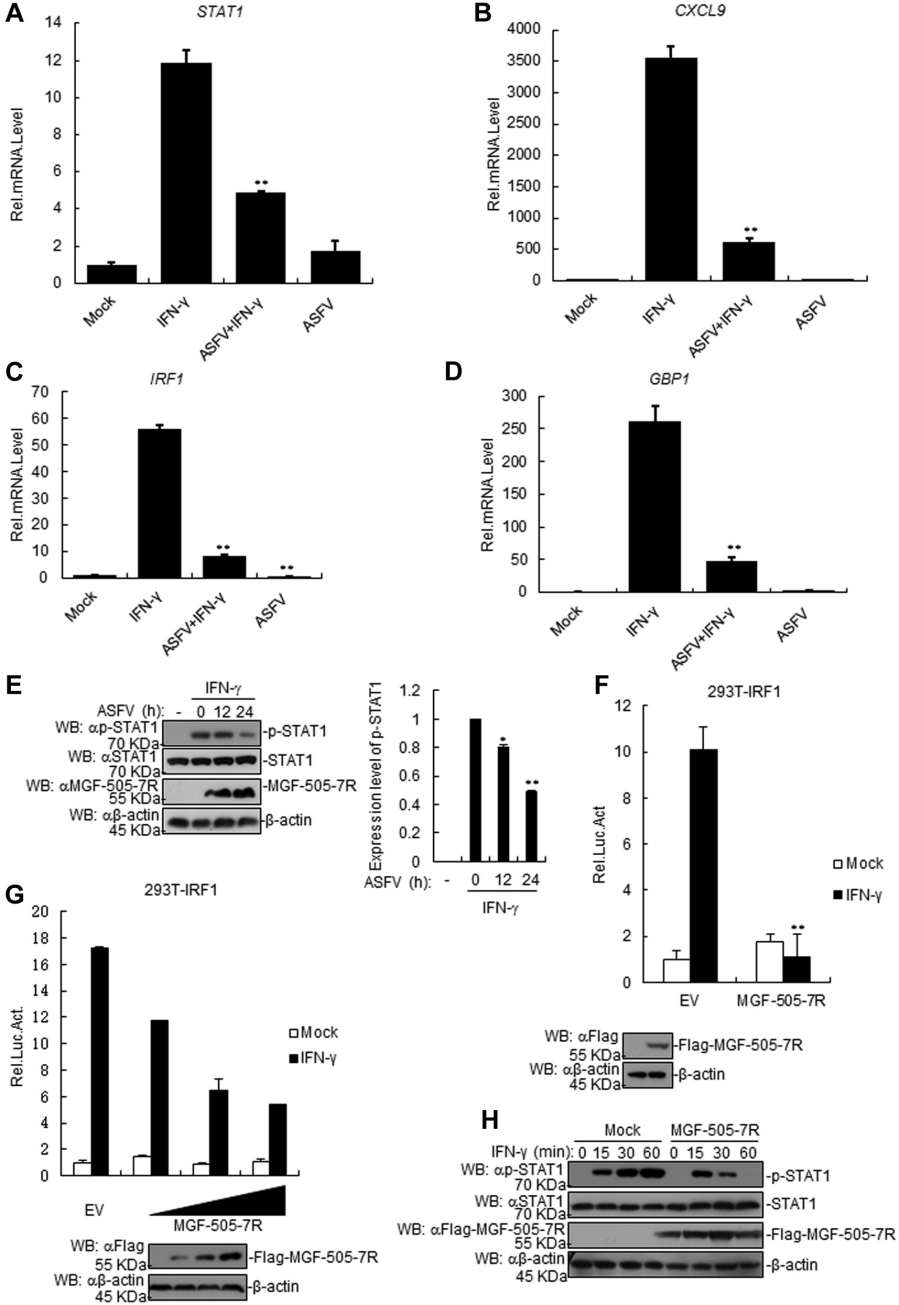
**The ASFV MGF-505-7R inhibits the IFN-γ-triggered signaling pathway.***A*–*D*, the effects of the ASFV on the IFN-γ-triggered transcription of *Stat1* (*A*), *Cxcl9* (*B*), *Irf1* (*C*), and *Gbp1* (*D*) genes in PAMs. PAMs were seeded into 12-well plates for 24 h. Cells were uninfected or infected with the ASFV (MOI: 0.01) for 36 h. Then, cells were treated or untreated with IFN-γ (10 ng/ml) for 3 h before the RT-PCR experiments. *E*, the ASFV inhibited the IFN-γ-induced phosphorylation of STAT1. PAMs were seeded into 12-well plates for 24 h. Cells were uninfected or infected with the ASFV (MOI: 0.01) for the indicated times. Then, cells were treated or untreated with IFN-γ (10 ng/ml) for 3 h. Cell lysates were analyzed using western blotting with the indicated antibodies. Densitometry quantification of p-STAT1 band was determined using ImageJ (*right*). *F*, the effects of the ASFV MGF-505-7R overexpression on the IFN-γ-triggered IRF1 promoter activation. First, 293T cells (1 × 10^5^) were transfected with the IRF1-luciferase reporter (0.1 μg) and the MGF-505-7R expression (0.1 μg) plasmids. Then, 20 h after transfection, the cells were treated or untreated with IFN-γ (10 ng/ml) for 12 h before luciferase assays were performed. The level of MGF-505-7R was analyzed using western blotting. *G*, dose-dependent effects of MGF-505-7R on the IFN-γ-triggered activation of the IRF1 promoter. The experiments were similarly performed as in *F*. *H*, MGF-505-7R expression inhibited the IFN-γ-induced phosphorylation of STAT1. MGF-505-7R-overexpressed 293T cells (2 × 10^5^) were treated or untreated with IFN-γ (10 ng/ml) for the indicated times. Meanwhile, the 293T cells were transfected with the empty vector as a control. Cell lysates were analyzed using western blotting with the indicated antibodies. Graphs show the mean ± SD, n = 3. Luc, luciferase.

To determine the effect of ASFV on IFN-γ-triggered signaling pathway, porcine alveolar macrophage (PAM) cells were infected with ASFV and treated with IFN-γ, and the transcription of downstream genes was analyzed by RT-PCR. The results showed that there was a higher level of *Stat1*, *Cxcl9*, *Irf1*, and *Gbp1* genetic transcription in IFN-γ-treated PAM cells than those of control cells (Fig. 1, *A*–*D*). Besides, we observed a significant decrease in the aforementioned gene transcription in IFN-γ-treated PAM cells post infection (Fig. 1, *A*–*D*), suggesting that IFN-γ-mediated signaling pathway was inhibited by ASFV infection. Moreover, ASFV infection reduced IFN-γ-triggered STAT1 phosphorylation in PAM cells in comparison with that in the control cells (Fig. 1*E*). These data suggest that ASFV negatively regulates IFN-γ-triggered signaling pathway.

### ASFV MGF-505-7R negatively regulates IFN-γ-triggered signaling

It has been shown that IFN-γ stimulation induces the expression of IFN-regulatory factor 1 (IRF1) gene whose promoter contains two conserved STAT1-binding sites (28). To identify the ASFV proteins that are involved in IFN-γ-triggered signaling regulation, 168 individual ASFV genes that could potentially inhibit IRF1 promoter were screened utilizing reporter assays in human embryonic kidney 293T (293T) cells. It was exhibited that ASFV MGF-505-7R inhibited the activation of the IRF1 promoter triggered by IFN-γ (Fig. 1*F*). Further experiments indicated that ASFV MGF-505-7R inhibited the IFN-γ-triggered activation of the IRF1 promoter in a dose-dependent manner (Fig. 1*G*). Moreover, overexpression of ASFV MGF-505-7R reduced the IFN-γ-triggered phosphorylation of STAT1 in 293T cells (Fig. 1*H*). These data suggest that ASFV MGF-505-7R protein negatively regulates IFN-γ-triggered signaling pathway.

### ASFV MGF-505-7R inhibited the transcription of a subset of IFN-γ-induced downstream genes

**Figure 2:**
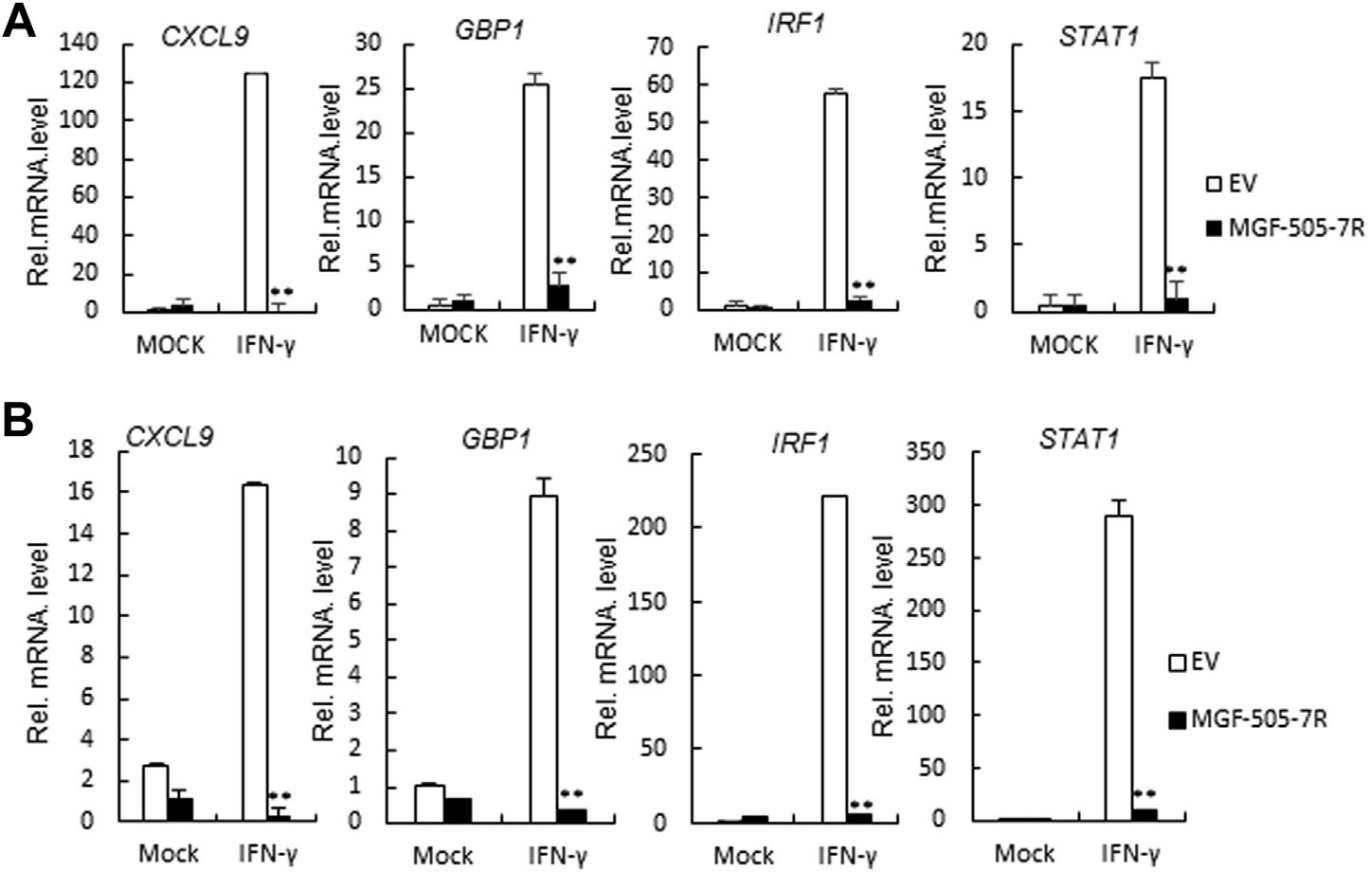
**The ASFV MGF-505-7R inhibits the expression of IFN-γ-triggered downstream antiviral genes.***A*, effects of the ASFV MGF-505-7R on the IFN-γ-triggered transcription of *Cxcl9*, *Gbp1*, *Irf1*, and *Stat1* genes. MGF-505-7R-overexpressed 293T cells (2 × 10^5^) were treated or untreated with IFN-γ (10 ng/ml) for 12 h before RT-PCR experiments were performed with the indicated primers. *B*, effects of the ASFV MGF-505-7R on the IFN-γ-triggered transcription of *Cxcl9*, *Gbp1*, *Irf1*, and *Stat1* genes in PAMs. The experiments were similarly performed as in *A*. Graphs show the mean ± SD, n = 3.

To investigate the influence of ASFV MGF-505-7R on IFN-γ-triggered type II IFN signaling pathway, we detected the transcription of a subset of IFN-γ-induced downstream genes in ASFV MGF-505-7R-overexpressed 293T cells by RT-PCR. The results showed that IFN-γ-triggered lower levels of the *Cxcl9*, *Gbp1*, *Irf1*, and *Stat1* transcripts in ASFV MGF-505-7R overexpressed 293T cells than those in the wild-type cells (Fig. 2*A*). The effects of ASFV MGF-505-7R on IFN-γ-triggered transcription of the *Cxcl9*, *Gbp1*, *Irf1*, and *Stat1* genes were not cell-type-specific because similar results were observed in PAM cells (Fig. 2*B*). These results suggest that ASFV MGF-505-7R inhibits IFN-γ-induced transcription of downstream genes.

### ASFV MGF-505-7R inhibits IFN-γ signaling pathway at JAK1 and JAK2 level

**Figure 3:**
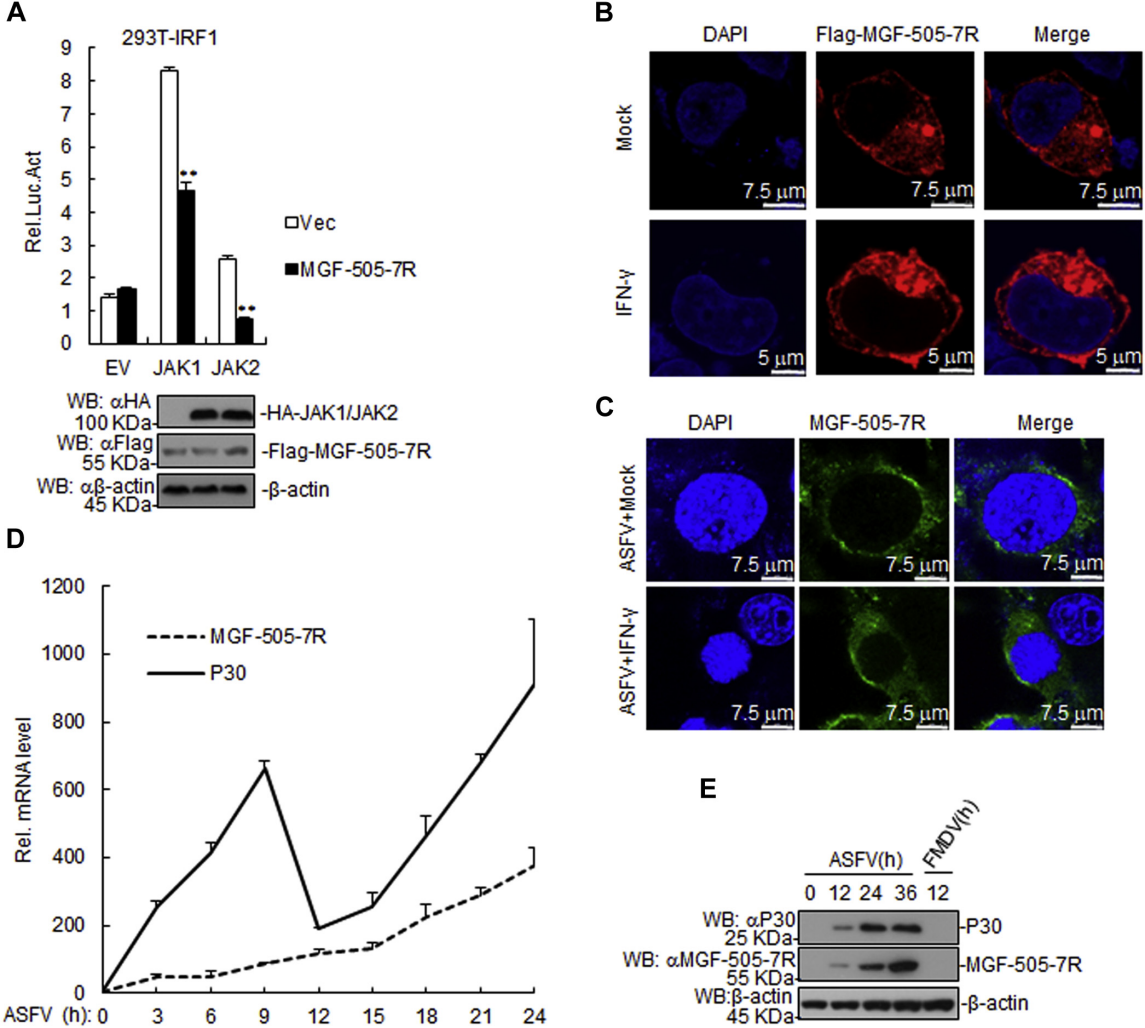
**The ASFV MGF-505-7R targets at the JAK1 and JAK2 levels.***A*, effects of MGF-505-7R on IRF1 activation by various signaling components. First, 293T cells (1 × 10^5^) were transfected with IRF1 reporter (0.1 μg) and expression plasmids for MGF-505-7R and the indicated plasmids (0.1 μg each) for 24 h. Then, luciferase assays were performed. The levels of MGF-505-7R, JAK1, and JAK2 were analyzed using western blotting. *B*, MGF-505-7R was located in the cytoplasm of the 293T cells. The 293T cells were transfected with expression plasmids encoding MGF-505-7R (1 μg). Then, 20 h after transfection, the cells were treated or untreated with IFN-γ (10 ng/ml) for 12 h before confocal microscopy. *C*, MGF-505-7R was located in the cytoplasm of the PAMs. PAMs were treated or untreated with IFN-γ (10 ng/ml) for 1 h. Then, the cells were uninfected or infected with the ASFV for 6 h and then fixed as well as stained with DAPI and anti-MGF-505-7R antibody. Finally, the location of MGF-505-7R in the cells was observed using a confocal microscope. *D*, the time course of MGF-505-7R gene transcriptional activity. The ASFV P30 MGF-505-7R open reading frame RNA prepared from *ex vivo* pig macrophages infected with the ASFV at the indicated times. *E*, detection of the specificity of the MGF-505-7R polyclonal antibody. PAMs were infected with the ASFV (MOI: 0.01) or FMDV (MOI: 0.1) for the indicated times. The samples were detected using western blotting with the indicated antibodies. Graphs show the mean ± SD, n = 3. Luc, luciferase.

Various components are involved in IFN-γ-triggered signaling pathways. As shown in Figure 3*A*, ASFV MGF-505-7R inhibited JAK1- and JAK2-triggered signaling pathways. In addition, we found that ASFV MGF-505-7R was localized in the cytoplasm of 293T cells upon transfection, while IFN-γ treatment did not alter its location (Fig. 3*B*). Moreover, we also found that MGF-505-7R was localized in the cytoplasm of the ASFV infected PAM cells, and its location did not change after IFN-γ treatment (Fig. 3*C*). In order to determine the kinetics of the MGF-505-7R transcripts production, we evaluated the total RNA extracted from the PAM cells infected with ASFV. The results showed that MGF-505-7R mRNA was expressed at early phase of infection as the same time as P30 mRNA, which is an ASFV gene expressed at early phase of infection, but was less abundant than P30 mRNA (Fig. 3*D*). In addition, to detect the specificity of MGF-505-7R polyclonal antibody, PAM cells were infected with ASFV or foot-and-mouth disease virus (FMDV). The result showed that ASFV P30 and MGF-505-7R proteins could be detected in ASFV-infected PAM cells but not in FMDV-infected PAM cells, suggesting that MGF-505-7R polyclonal antibody specifically binds ASFV MGF-505-7R protein (Fig. 3*E*). These results suggest that ASFV MGF-505-7R inhibits IFN-γ signaling pathway at JAK1 and JAK2 level.

### ASFV MGF-505-7R interacts with JAK1 and JAK2

**Figure 4:**
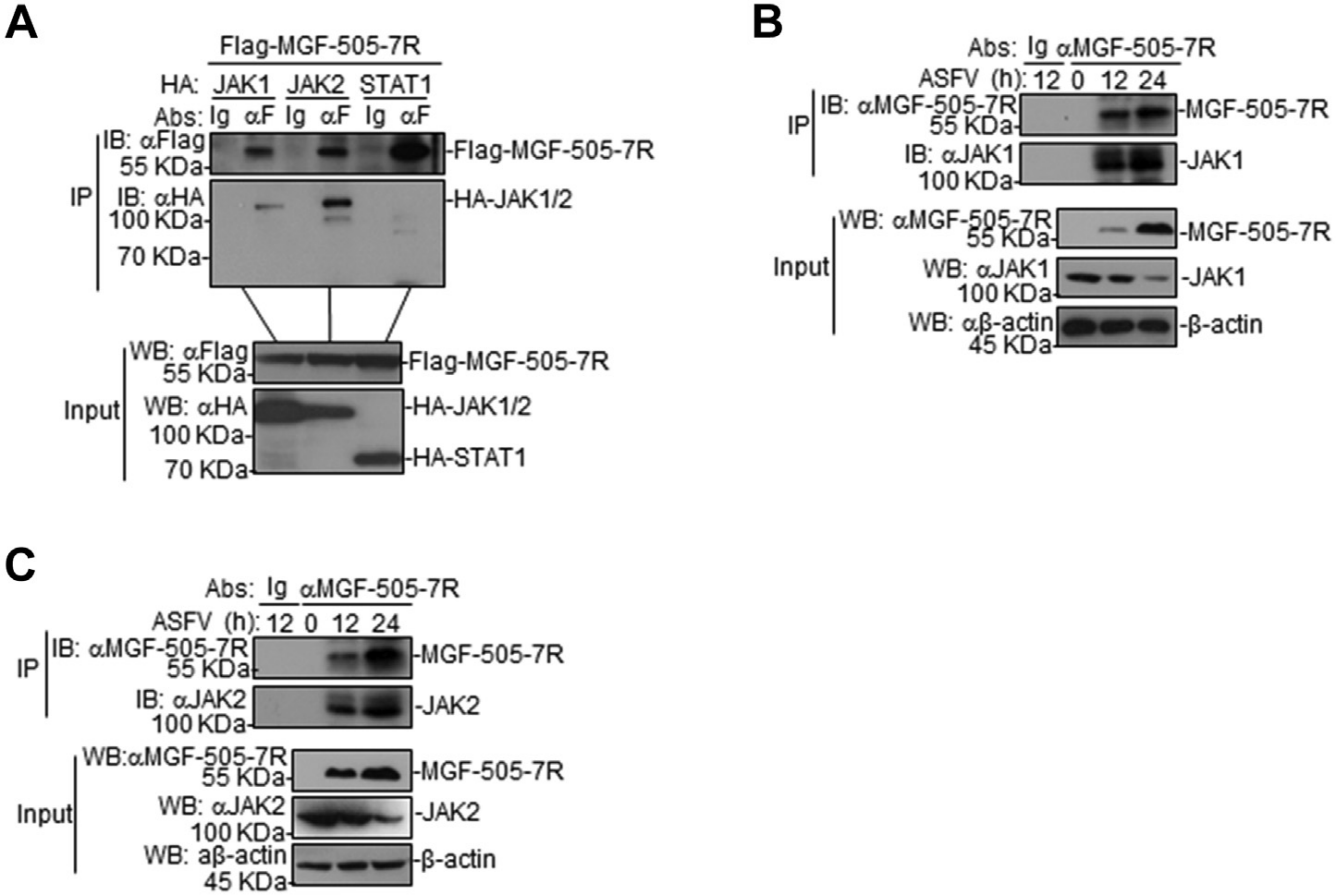
**The ASFV MGF-505-7R interacts with JAK1 and JAK2.***A*, MGF-505-7R interacted with JAK1 and JAK2 but not with STAT1 in the overexpression system. First, 293T cells (2 × 10^6^) were transfected with the indicated plasmids (5 μg each). Coimmunoprecipitation and western blot analyses were performed with the indicated antibodies. The levels of the transfected proteins were analyzed using western blotting with anti-HA or -Flag antibodies. *B* and *C*, interactions between the endogenous MGF-505-7R and JAK1 and JAK2. PAMs were uninfected or infected with the ASFV for the indicated times before coimmunoprecipitation and western blot analyses.

To determine whether ASFV MGF-505-7R is associated with IFN-γ signaling components, coimmunoprecipitation experiments were performed in 293T cells. The results showed that ASFV MGF-505-7R interacted with JAK1 and JAK2, but not with STAT1 (Fig. 4*A*). To determine whether it was the same case in the virus-infected PAM cells, cell lysates were immunoprecipitated with an anti-MGF-505-7R polyclonal antibody and probed for the presence of JAK1 and JAK2 with anti-JAK1 and -JAK2 polyclonal antibodies, respectively. JAK1 and JAK2 were readily detected in the ASFV-infected PAM cells (Fig. 4, *B* and *C*), indicating that MGF-505-7R indeed interacted with endogenous JAK1 and JAK2 proteins in the infected PAM cells. Collectively, these findings confirm that JAK1 and JAK2 interact with ASFV MGF-505-7R protein.

### ASFV MGF-505-7R degrades JAK1 and JAK2 proteins

**Figure 5:**
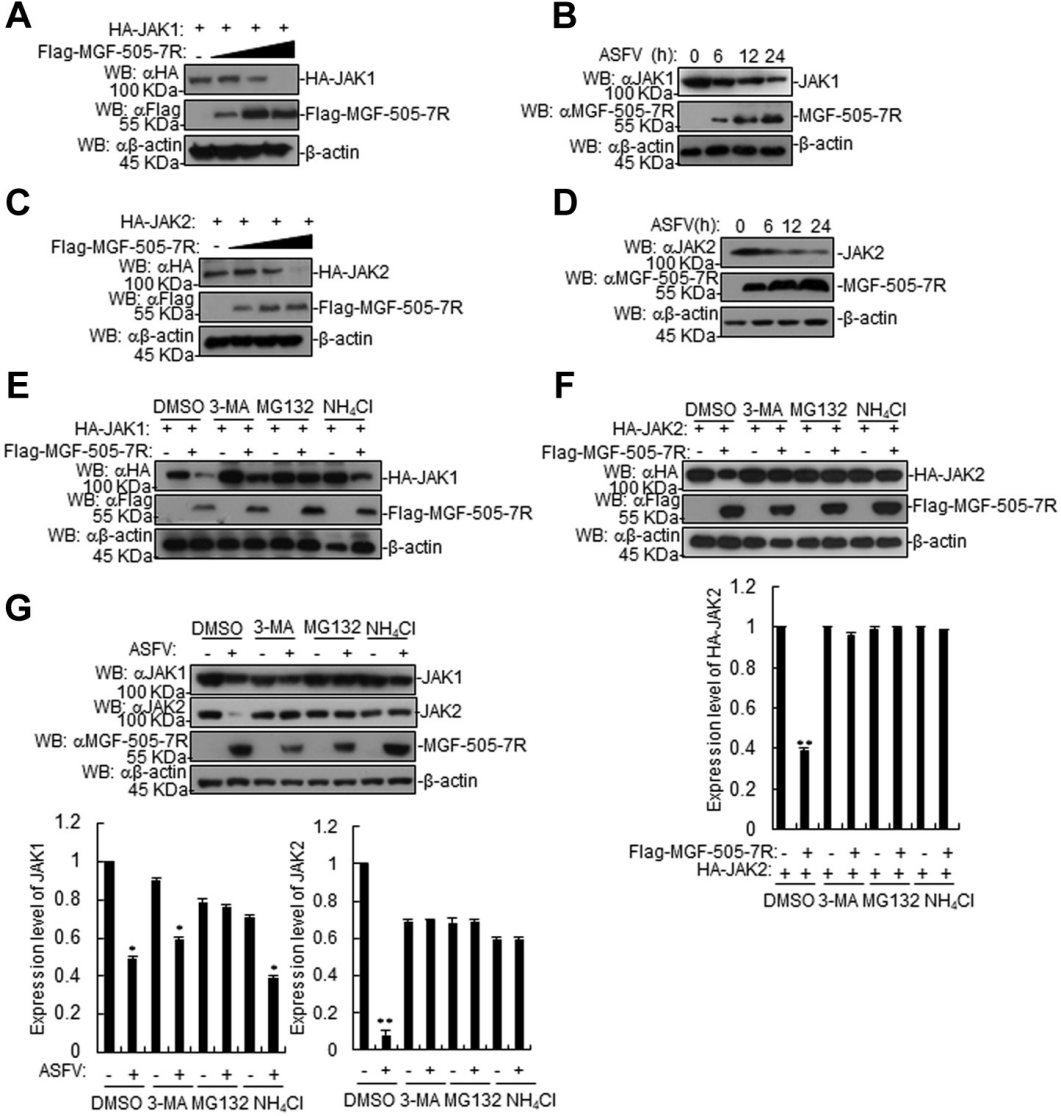
**The ASFV MGF-505-7R degrades JAK1 and JAK2.***A*, the effects of the ASFV MGF-505-7R on the expression of JAK1 in 293T cells. The 293T cells (2 × 10^5^) were transfected with HA-JAK1 (1 μg) and Flag-MGF-505-7R (0, 0.25, 0.5, and 1.0 μg) for 24 h. Cell lysates were analyzed using western blotting with the indicated antibodies. *B*, the effects of the ASFV on the endogenous JAK1 in PAMs. PAMs were uninfected or infected with the ASFV for the indicated times and were then analyzed using western blotting with the indicated antibodies. *C*, effects of the ASFV MGF-505-7R on the expression of JAK2 in 293T cells. The experiments were similarly performed as in *A*. *D*, the effects of ASFV infection on endogenous JAK2 in PAMs. The experiments were similarly performed as in *B*. *E*, the effects of inhibitors on the MGF-505-7R-mediated destabilization of JAK1. The 293T cells (4 × 10^5^) were transfected with the indicated plasmids. Then, 18 h after transfection, the cells were treated with the indicated inhibitors for 6 h before western blotting was performed. *F*, the effects of inhibitors on the MGF-505-7R-mediated destabilization of JAK2. The experiments were similarly performed as in *E*. Densitometry quantification of JAK2 band was determined using ImageJ (down). *G*, the effects of inhibitors on the ASFV-mediated destabilization of JAK1 and JAK2. The PAMs were infected with the ASFV (MOI: 0.01) for 18 h. The cells were treated with MG132 (100 μM), NH_4_Cl (25 mM), or 3-MA (500 ng/ml) for 6 h before western blotting was performed. Densitometry quantification of JAK1 and JAK2 bands was determined using ImageJ (down). Graphs show the mean ± SD, n = 3.

We next investigated how ASFV MGF-505-7R regulates JAK1 and JAK2 in IFN-γ-triggered signaling. 293T cells were cotransfected with plasmids containing MGF-505-7R and either JAK1 or JAK2 genes to test whether MGF-505-7R affects the expression of JAK1 and JAK2. Western blotting revealed that overexpression of MGF-505-7R decreased JAK1 and JAK2 protein levels (Fig. 5, *A* and *C*). To explore the effect of ASFV infection on JAK1 and JAK2 expression, PAM cells were infected with ASFV for indicated time courses and subjected to respective antibody detections. The results showed that ASFV infection also inhibited the expression of JAK1 and JAK2 proteins in PAMs (Fig. 5, *B* and *D*). Protein degradation is one of the main strategies that viruses utilize to impair host cellular protein functions during infection *via* at least three routes, including the ubiquitin-proteasome, lysosomal, and autophagosome pathways. To begin dissecting the mechanisms responsible for MGF-505-7R influencing the stability of JAK1 and JAK2, we transfected 293T cells with the indicated plasmids and treated the cells with various inhibitors for protein degradation pathways. MGF-505-7R- or ASFV-mediated degradation of JAK1 was completely inhibited by the proteasome inhibitor MG132, but not lysosomal inhibitor NH_4_Cl or autophagosome inhibitor 3-MA (Fig. 5, *E* and *G*). We also found that JAK2 degradation mediated by MGF-505-7R or ASFV was absolutely inhibited by NH_4_Cl, 3-MA or MG-132 (Fig. 5, *F* and *G*). These results suggest that MGF-505-7R could inhibit the IFN-γ-triggered signaling pathway by degrading JAK1 and JAK2 proteins.

### MGF-505-7R degrades JAK1 by upregulating RNF125 expression and JAK2 by downregulating Hes5 expression

**Figure 6:**
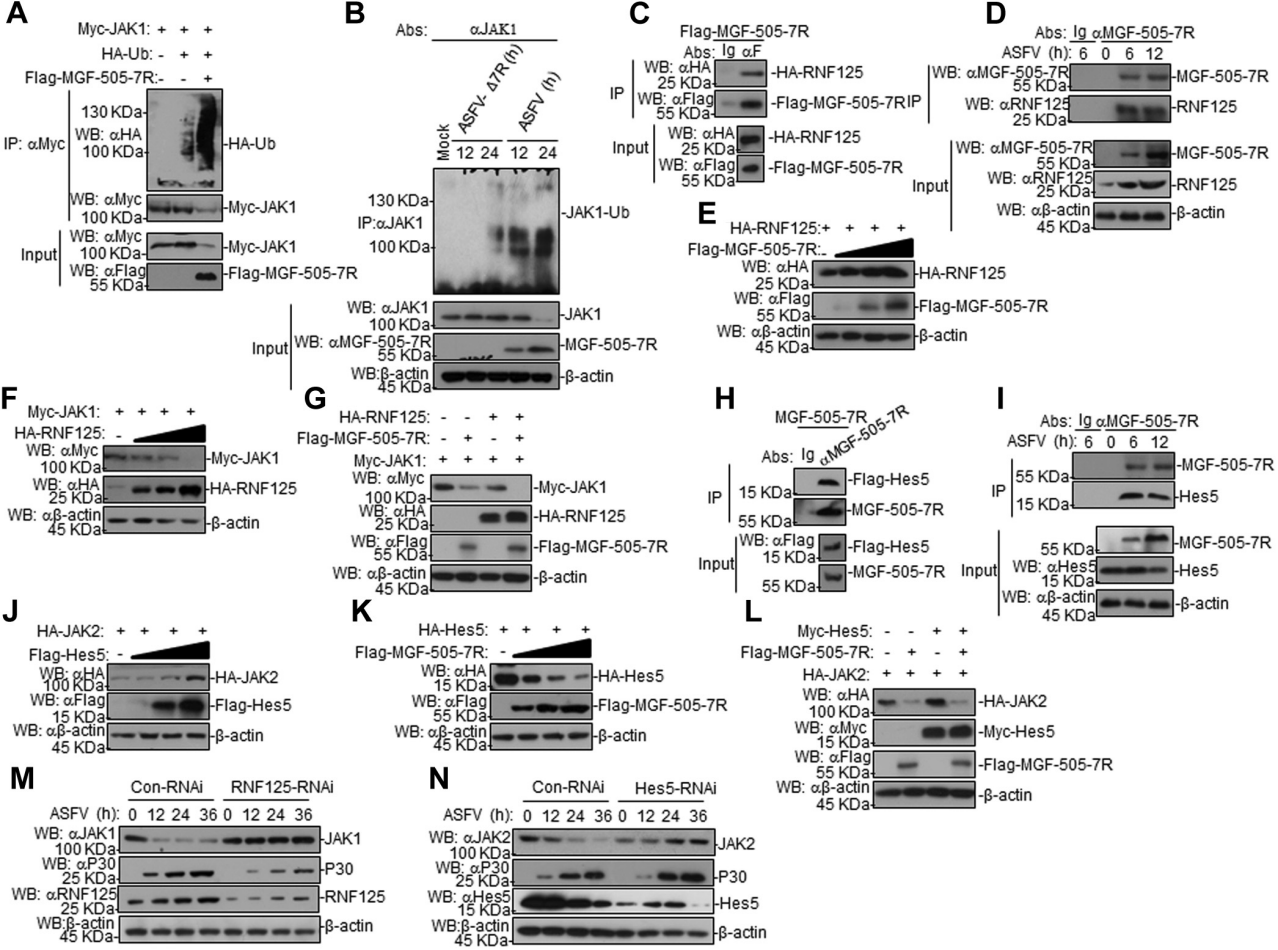
**The ASFV MGF-505-7R degrades JAK1 by upregulating RNF125 expression and JAK2 by downregulating Hes5 protein.***A*, MGF-505-7R enhanced JAK1 ubiquitination. First, 293T cells (2 × 10^5^) were transfected with Myc-JAK1 (1 μg), HA-Ub (0.5 μg), and Flag-MGF-505-7R (1 μg) for 24 h. Coimmunoprecipitation and western blot analyses were performed with the indicated antibodies. *B*, coimmunoprecipitation and western blot analyses for the detection of the endogenous polyubiquitination of JAK1 in the ASFV- or ASFV-Δ7R-infected PAMs for the indicated times. PAMs were infected with the ASFV or ASFV-Δ7R (MOI: 0.01) for the indicated times. Coimmunoprecipitation and western blot analyses were performed with the indicated antibodies. *C*, MGF-505-7R interacted with RNF125 in the overexpression system. The 293T cells (2 × 10^6^) were transfected with the indicated plasmids (5 μg each). Coimmunoprecipitation and western blot analyses were performed with the indicated antibodies. The levels of the transfected proteins were analyzed using western blotting with anti-HA or -Flag antibodies. *D*, endogenous associations between MGF-505-7R and RNF125. PAMs were infected with the ASFV for the indicated times before coimmunoprecipitation and western blot analyses. *E*, MGF-505-7R increased the expression of the RNF125 protein. First, 293T cells (2 × 10^5^) were transfected with HA-RNF125 (1 μg) and Flag-MGF-505-7R (0, 0.25, 0.5, and 1.0 μg) for 24 h. Cell lysates were analyzed using western blotting with the indicated antibodies. *F*, RNF125 inhibited the expression of JAK1 protein. The experiments were similarly performed as in *E*. *G*, RNF125 increased the MGF-505-7R degradation of JAK1. The 293T cells (2 × 10^5^) were transfected with the indicated plasmids for 24 h. Cell lysates were analyzed using western blotting with the indicated antibodies. *H*, MGF-505-7R interacted with Hes5. The experiments were similarly performed as in *A*. *I*, endogenous associations between MGF-505-7R and RNF125. The experiments were similarly performed as in *D*. *J*, Hes5 increased the expression of JAK2 protein. The experiments were similarly performed as in *E*. *K*, MGF-505-7R inhibited the expression of Hes5 protein. The experiments were similarly performed as in *E*. *L*, MGF-505-7R inhibited the Hes5-mediated expression of JAK2. The experiments were similarly performed as in *G*. *M* and *N*, the effect of RNF125 or Hes5 knockdown on JAK1 or JAK2 expression. The PAMs were transfected with Con-RNAi, RNF125-RNAi, or Hes5-RNAi (1.0 μg/ml) for 48 h. Then, the cells were infected with the ASFV (MOI: 0.01) for the indicated time. Cell lysates were analyzed using western blotting with the indicated antibodies. Con-RNAi, control-RNAi.

The above results showed that MGF-505-7R degraded JAK1 by the proteasome pathway; thus, we next examined whether MGF-505-7R expression increased in JAK1 ubiquitination and, if so, which E3 ubiquitin ligase mediated MGF-505-7R degradation of JAK1. In the transient transfection and coimmunoprecipitation experiments, we discovered that MGF-505-7R and ASFV increased JAK1 ubiquitination, but ASFV-Δ7R inhibited JAK1 ubiquitination compared with ASFV (Fig. 6, *A* and *B*). A previous study reported that JAK1 interacts with RNF125 (29); thus, we investigated whether RNF125 mediated MGF-505-7R degradation of JAK1. To test this possibility, we performed coimmunoprecipitation by transiently cotransfecting 293T cells with Flag-tagged ASFV MGF-505-7R and HA-tagged RNF125 plasmids. Both 293T cell cotransfection and infection studies in PAM cells showed that ASFV MGF-505-7R interacted with RNF125 (Fig. 6, *C* and *D*). We also found that MGF-505-7R increased the expression of RNF125 in 293T cells in a dose-dependent manner, while RNF125 inhibited the expression of JAK1 (Fig. 6, *E* and *F*). In addition, we found that RNF125 increased MGF-505-7R degradation of JAK1 (Fig. 6*G*). These data collectively indicated that MGF-505-7R degrades JAK1 by upregulating RNF125 expression.

It has been reported that Hes5 interacts with JAK2 and increases its expression (30). To test whether MGF-505-7R interacts with Hes5, we implemented coimmunoprecipitation and found that MGF-505-7R was associated with Hes5 in 293T cells (Fig. 6*H*) and ASFV-infected PAM cells (Fig. 6*I*). To further investigate the role of Hes5 in the expression of JAK2, we transfected 293T cells with expression plasmids encoding the adaptor protein JAK2 together with different amounts of Hes5 and observed that the expression of JAK2 was enhanced by Hes5 in a dose-dependent manner (Fig. 6*J*), whereas Hes5 expression was inhibited by MGF-505-7R in a dose-dependent manner (Fig. 6*K*). Furthermore, it was shown that Hes5-mediated JAK2 expression was impeded by MGF-505-7R (Fig. 6*L*). Next, to explore the effect of RNF125 or Hes5 on JAK1 or JAK2 respectively, we synthesized small interfering RNAs targeting RNF125 or Hes5. The result showed that the expression of JAK1 or JAK2 was increased in RNF125- or Hes5-knockdown PAM cells infected with ASFV (Fig. 6, *M* and *N*). These data collectively demonstrate that MGF-505-7R inhibited the expression of JAK2 *via* downregulating the expression of Hes5.

### MGF-505-7R-deficient ASFV increased type II IFN signaling pathway

**Figure 7:**
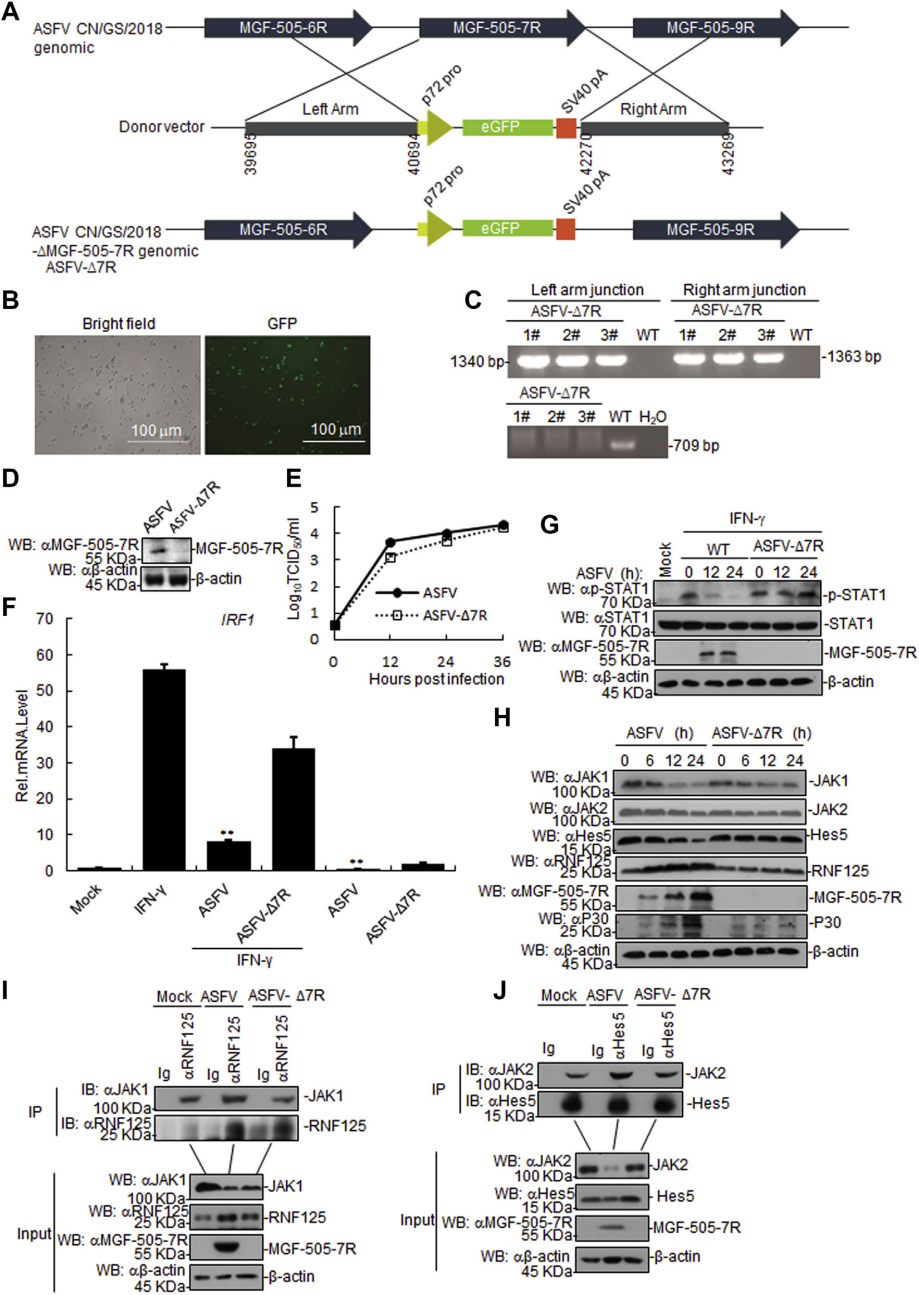
**The MGF-505-7R-deficient ASFV increased the JAK-STAT1 signaling pathway.***A*, diagram indicating the position of the MGF-505-7R open reading frame in the ASFV CN/GS/2018 genome. The donor plasmid with the homologous arms to the ASFV CN/GS/2018 and eGFP under control of the p72 promoter in the orientation as indicated. The final genomic changes introduced to develop the ASFV-Δ7R where the sequence of the donor plasmid eGFP reporter was introduced to replace the ORF of the MGF-505-7R as indicated. *B*, the successful recombination was confirmed using a fluorescence microscope. *C*, the absence of parental CN/GS/2018 was confirmed using PCR. *D*, effects of the ASFV MGF-505-7R knockout (ASFV-Δ7R) on the expression of the endogenous ASFV MGF-505-7R. *E*, replication of the ASFV-Δ7R in Ma-104 cells. Ma-104 cells were infected with the parental ASFV or ASFV-Δ7R (MOI: 0.01) for the indicated time before RT-PCR analysis. Data represent the means from three independent experiments. *F*, effects of the ASFV or ASFV-Δ7R on IFN-γ-induced *Irf1* transcription. PAMs were infected with the parental ASFV or ASFV-Δ7R (MOI: 0.01) for 21 h. The cells were untreated or treated with IFN-γ (10 ng/ml) for 3 h before RT-PCR analysis. Data represent the means from three independent experiments. *G*, effects of the ASFV-Δ7R on the IFN-γ-induced phosphorylation of downstream components. PAMs were infected with the parental ASFV or ASFV-Δ7R for the indicated times and were then treated or untreated with IFN-γ for 3 h. *H*, effects of ASFV-Δ7R on JAK1 and JAK2, Hes5, P30, and RNF125 expression. The cells were infected with the parental ASFV or ASFV-Δ7R for the indicated times before western blot analysis. *I*, effects of the ASFV or ASFV-Δ7R on the interaction between RNF125 and JAK1. PAMs were infected with the ASFV or ASFV-Δ7R (MOI: 0.01) for 12 h before coimmunoprecipitation and western blot analyses. *J*, effects of the ASFV or ASFV-Δ7R on the interaction between Hes5 and JAK2. The experiments were similarly performed as in *I*.

In order to investigate the function of endogenous ASFV MGF-505-7R in JAK-STAT1 signaling, we generated an MGF-505-7R-deficient virus (namely as ASFV-Δ7R) by genetic modification of the highly virulent ASFV CN/GS/2018 strain using CRISPR/Cas9 system. The *MGF-505-7R* gene was replaced by a cassette encoding the fluorescent eGFP under the control of ASFV p72 promoter (Fig. 7*A*). The mutant virus was purified *via* 11 rounds of limited dilutions by selecting eGFP-positive clones (Fig. 7*B*). The absence of *MGF-505-7R* gene in the ASFV-ΔMGF-505-7R virus was confirmed by PCR (Fig. 7*C*). Western blotting confirmed that MGF-505-7R was undetectable in ASFV-Δ7R infected PAM cells (Fig. 7*D*). To evaluate the replication of ASFV-Δ7R in nonimmune cells, Ma-104 cells that are susceptible to ASFV (31) were infected with ASFV and ASFV-Δ7R. The results showed that the replication of ASFV-Δ7R virus was same as the parental ASFV in Ma-104 cells (Fig. 7*E*). We observed that the *Irf1* transcripts were higher in ASFV-Δ7R infected PAM cells than in cells infected with parental ASFV (Fig. 7*F*). Consistently, IFN-γ-induced *Irf1* transcripts were higher in ASFV-Δ7R infected PAM cells than cells infected with parental ASFV (Fig. 7*F*). It was consistent with the replication kinetics that the ASFV-Δ7R increased IFN-γ-induced the p-STAT1 level compared with parental ASFV (Fig. 7*G*). As expected, we also observed that ASFV-Δ7R infection elevated the JAK1, JAK2, and Hes5 expression level in PAM cells, but not the RNF125 and ASFV p30 expression level in comparison with those of parental ASFV-infected cells (Fig. 7*H*). Additionally, we found that the interaction between RNF125 and JAK1 was increased in ASFV-infected PAM cells compared with that of the control, but the interaction was inhibited in ASFV-Δ7R infected PAM cells compared with that of the parental ASFV-infected PAM cells (Fig. 7*I*). Coimmunoprecipitation experiments showed that the interaction between Hes5 and JAK2 increased in the ASFV but not in the ASFV-Δ7R-infected PAM cells (Fig. 7*J*). Collectively, these results suggest that ASFV-Δ7R infection enhances the type II IFN signaling in comparison with parental ASFV.

### Assessment of ASFV-Δ7R virulence in swine

**Figure 8:**
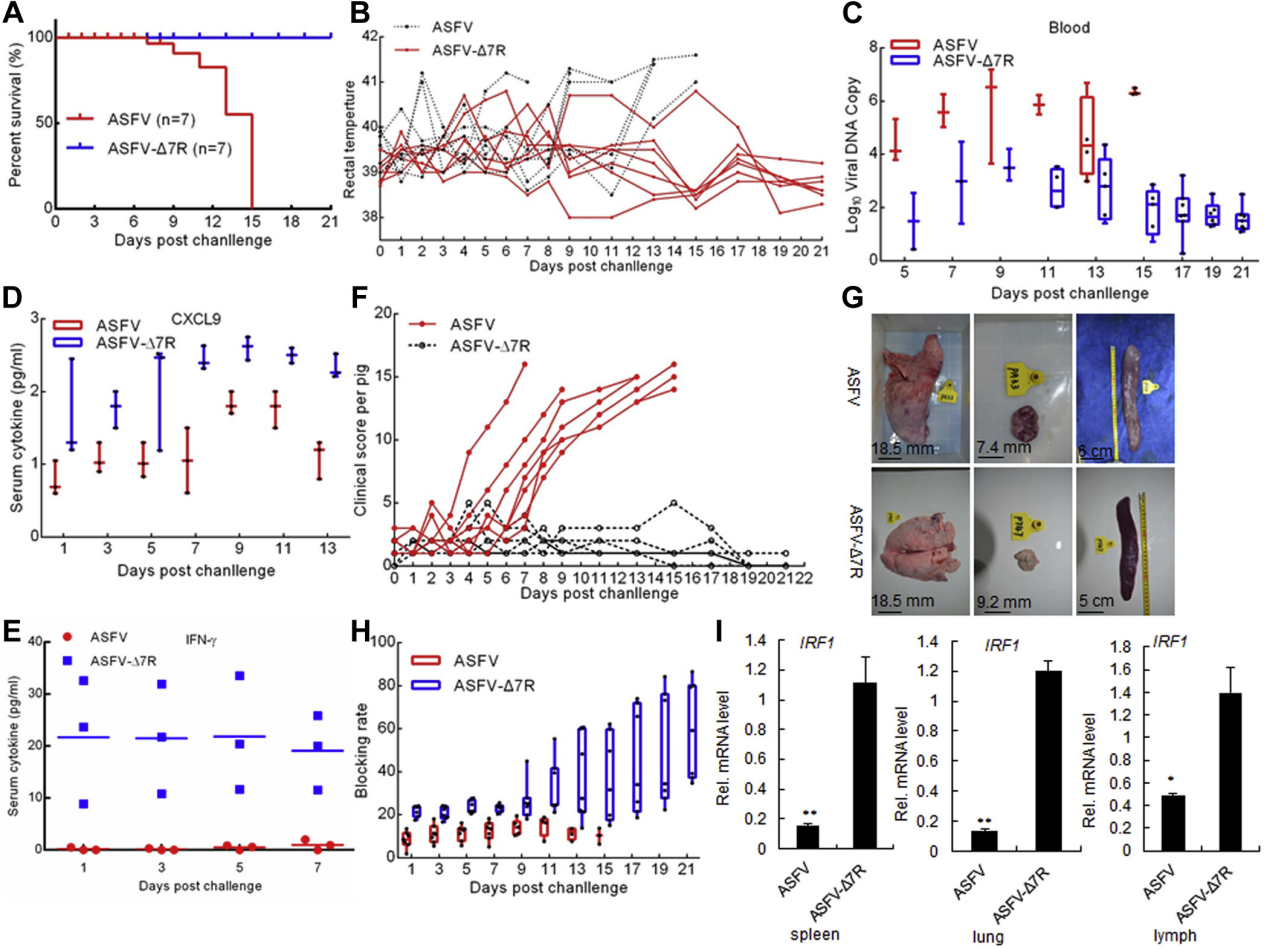
**Assessment of the ASFV-Δ7R virulence in swine.** Pigs were inoculated intramuscularly (i.m.) with either ten HAD_50_ of ASFV-ΔMGF-505-7R (n = 7) or ASFV-WT (n = 7). The pigs were monitored daily for 21 days for temperature and mortality. *A*–*E*, (*A*) Survival rates; (*B*) Daily temperature changes of the pigs; (*C*) Numbers of viral DNA copies in blood collected at the indicated days postchallenge; (*D*) The expression level of CXCL9 (*E*) or IFN-γ in serum at the indicated days postchallenge, determined using ELISA. *F*, the clinical score of individual pigs infected with the ASFV or ASFV-Δ7R. *G*, gross pathological changes of the spleen, lungs, and lymph nodes in the two groups of pigs. Severe, enlarged, friable, and dark black appearance of the spleen infected with the ASFV. Mild interstitial edema of the lung and mildly enlarged and red-black appearance of the lymph nodes in pigs infected with the ASFV. *H*, the levels of antibodies against p30 were determined using an in-house blocking ELISA. *I*, effects of ASFV-Δ7R on the IRF1 transcription level induced by i.m. inoculation with the parental ASFV or ASFV-Δ7R (ten HAD_50_ for each pig) for 6 days (n = 3 per group).

To investigate whether ASFV-Δ7R was attenuated in pigs, we intramuscularly injected pigs with ten 50% hemadsorption (HAD) doses (HAD_50_) of the gene-deleted virus or parental ASFV and observed the pigs for 3 weeks. The results showed that all of the pigs inoculated with parental ASFV died within 15 days, whereas those inoculated with ASFV-Δ7R all survived and remained healthy throughout the 3-week observation period (Fig. 8*A*). All seven pigs inoculated with wild-type ASFV developed symptoms of fever with maximum temperature up to 41.6 °C (Fig. 8*B*). Pigs inoculated with the ASFV-ΔMGF-505-7R virus displayed moderate body temperature increase and less severe symptoms, and all seven pigs survived from the infection (Fig. 8*B*). Pigs infected with the ASFV-Δ7R virus had much lower viral loads in blood than pigs infected with wild-type ASFV (Fig. 8*C*). Pigs infected with ASFV-Δ7R virus produced higher levels of serum CXCL9 and IFN-γ than those infected with wild-type ASFV at days postinfection (Fig. 8, *D* and *E*). Previous studies showed that the clinical symptoms of experimental animals infected with ASFV were comprehensively evaluated by the clinical score standard (32). The results in this study showed that the clinical score of pigs infected with ASFV was significantly higher than that of pigs infected with ASFV-Δ7R (Fig. 8*F*). The pathological changes of gross organs in pigs injected with ASFV were significantly more serious than those in pigs injected with ASFV-Δ7R (Fig. 8*G*). Previous studies have indicated that the levels of circulating antibodies, such as the antibody against p30, are important parameters consistently associated with humoral immune responses (33). We found that the surviving pigs infected with the ASFV-ΔMGF-505-7R virus exhibited increased levels of p30 antibody at the late stage of infection (Fig. 8*H*), indicating that the attenuated ASFV-ΔMGF-505-7R virus induced better antibody responses. The IRF1 transcription level in various tissues derived from ASFV-Δ7R-infected pigs was higher than that of the parental ASFV-infected counterparts (Fig. 8*I*). Collectively, these data suggest that ASFV-Δ7R is significantly attenuated in pigs and is completely nonlethal to swine at ten HAD_50_.

## Discussion

Pathogenic ASFV genomes contain 11 to 15 multigene family 360 (MGF360) genes and either nine or ten MGF530 (MGF505) genes (15). It is widely accepted that gene-deleted viruses lacking members of MGF 360 and 505/530 are partially sensitive to type I IFN pretreatment, suggesting that these genes play a role in inhibiting the IFN responses or antiviral state (14). The ASFV MGF-505-7R is a member of MGF505; thus, it could potentially serve as an IFN evasion protein. In the study, we found that MGF-505-7R inhibited IFN-γ-triggered signaling pathway *via* degradation of JAK1 and JAK2.

Although the inhibition of cellular responses to type I or II IFN is a common strategy that viruses utilize for immune evasion, it is surprising that no ASFV gene has been well described to inhibit the JAK-STAT signaling cascade, which is the central transduction pathway mediating IFN antiviral effects (13). To our knowledge, our study is the first to describe the mechanism of how ASFV MGF-505-7R protein inhibits the IFN-γ-triggered signaling pathway.

To date, several studies have demonstrated that ASFV evades the host innate immune responses through regulating type I IFNs (14, 15, 16). Nevertheless, the role of IFN-γ signaling pathway in ASFV evading host’s innate immune responses remains unclear. It is well known that the majority of type II IFN expression is induced by invading pathogens (13). Type II IFN is produced both in the early stages of infection by NK cells or macrophages (34, 35) and at later stages by activated T lymphocytes (36), either *via* receptor-mediated stimulation (through T or NK cell receptors) or in response to early produced cytokines, such as IL-12, IL-18, and IFN-α/β (35). Although other cells can be infected at later phase of infection, ASFV primarily replicates in monocytes and macrophages at early stage of infection (37, 38). On the basis of the current findings, we speculated that there was a correlation between ASFV infection and type II IFN response.

JAK1 and JAK2 play an important role in IFN-γ-triggered signaling pathway. On the one hand, tyrosine kinase JAK1 and JAK2 are associated with IFN-γ receptors (IFNGR1 and IFNGR2), and on the other hand, JAKs directly phosphorylate STAT1 at Tyr701 and indirectly lead to its phosphorylation at Ser727, which activate the IFN-γ-triggered signaling pathway. In this aspect, we found that MGF-505-7R degraded JAK1 *via* upregulating RNF125 expression and JAK2 expression by downregulating Hes5 to inhibit the IFN-γ-triggered signaling pathway.

One of the major obstacles in ASF disease control is the unavailability of a suitable commercialized vaccine. The use of attenuated strains is currently the most plausible approach to develop an effective ASF vaccine. Rational development of attenuated strains by genetic manipulation is a valid alternative and perhaps a safer methodology compared with the utilization of naturally attenuated isolates. Deletions of single genes or a group of genes by genetic manipulation have been shown to an effective way of producing attenuated strains to induce protection against the virulent parental virus challenges (39, 40, 41, 42, 43, 44, 45). Here, we documented that deletion of ASFV MGF-505-7R significantly attenuated the virus in swine. Deletion of the 9GL gene (particularly potentiated by the additional deletion of the UK gene), deletion of a group of six genes from the MGF360 and 530, or deletion of I177L has been shown to completely abolish virulence in the highly virulent ASFV Georgia isolate (41, 44, 45, 46). As ASFV-G has not been efficiently attenuated by deletion of other genes except for I177L gene that has been associated with attenuation in other ASFV isolates (41, 44, 47), the attenuation observed by deleting the MGF-505-7R gene is an important discovery. Several previous studies have shown that generating attenuated ASFV recombinant viruses by genetic manipulations is an effective approach for vaccine development.

In summary, our findings show that the ASFV MGF-505-7R antagonizes the JAK-STAT1 axis by impairing IFN-γ-mediated activation of JAK1 and JAK2 and therefore, acts as a critical determinant of the virulence of ASFV. Our study provides important clues for the mechanisms of immune evasion by large DNA viruses. Identification of MGF-505-7R as an important inhibitor of innate immune responses and critical determinant of its virulence points provide the possibility of developing MGF-505-7R-deficient attenuated vaccines for ASFV.

## Experimental procedures

### Cell culture and virus infections

PAM was prepared by bronchoalveolar lavage as previously described (48) and grown in DMEM supplemented with 2 mM L-glutamine, 100 U/ml gentamicin, nonessential amino acids, and 10% porcine serum. 293T cells and Ma-104 cells were cultivated in DMEM supplemented with 10% fetal bovine serum, 100 U penicillin/ml, and 100 μg streptomycin/ml. All cells were grown at 37 °C in a 5% CO_2_ atmosphere saturated with water vapor. The ASFV isolates CN/GS/2018 (49) were propagated on PAM cells as previously described (17). The type O FMDV was propagated in PK-15 cells, and the supernatants of the infected cells were clarified and stored at −80 °C.

### Constructs

IRF1 promoter luciferase reporter plasmids and mammalian expression plasmids for HA-tagged JAK1, JAK2, STAT1, Ub, and Myc-JAK1 were previously described (50). Mammalian expression plasmids for Flag-tagged Hes5 (XM_021097834.1) and HA-tagged RNF125 (XM_003127888.5) were constructed by standard molecular biology techniques. For construction of Flag-MGF-505-7R, the DNA fragment containing the full-length MGF-505-7R of ASFV isolate CN/GS/2018 was amplified by PCR from the cDNA of ASFV and subcloned into pRK-Flag vector.

### Antibodies and reagents

Rabbit anti-MGF-505-7R and anti-P30 sera were raised against a recombinant ASFV MGF-505-7R and P30 proteins. Polyclonal rabbit anti STAT1 (#14994), phospho-STAT1 (Tyr701) (#9167), JAK1 (#29261), and JAK2 (#3230) were purchased from Cell Signaling Technology. Polyclonal rabbit anti RNF125 (ab211450) and Hes5 (ab194111) were purchased from Abcam. Monoclonal mouse anti HA (H3663), β-actin (A5441), Myc (SAB1305535), and Flag (F3040) were purchased from Sigma-Aldrich. Alexa Fluor-488-conjugated goat anti-mouse IgG (H + L) and Alexa Fluor 594-conjugated goat anti-rabbit IgG (H + L) antibodies were purchased from Cell Signaling Technology. 3-MA (189490) and MG132 (M8699) were purchased from Sigma-Aldrich. Porcine IFN-γ ELISA kit was purchased from Solarbio Life Sciences (SEKP-0010).

### Transfection and reporter gene assays

293T cells (1 × 10^5^) were seeded on 24-well plates and transfected on the following days by standard calcium phosphate precipitation method. In the same experiment, empty control plasmid was added to ensure that each transfection receives the same amount of total DNA. To normalize for transfection efficiency, 0.01 μg of pRL-TK Renilla luciferase reporter plasmid was added to each transfection. Luciferase assays were performed using a dual-specific luciferase assay kit (Promega), and the firefly luciferase activities were normalized based on Renilla luciferase activities.

### Cell lines and retroviral gene transfer

Transduction of MGF-505-7R to PAM cells was performed by retroviral-mediated gene transfer. Briefly, 293T cells plated on 100-mm dishes were transfected with the indicated retroviral expression plasmid (10 μg) together with the pGag-pol (10 μg) and the pVSV-G (3 μg) plasmids. Two days after transfection, the viruses were harvested and used to infect the indicated cells in the presence of polybrene (4 μg/ml). The infected PAM cells were selected with puromycin (2 μg/ml) for 12 h.

### Confocal microscopy

293T cells were transfected with Flag-MGF-505-7R (1 μg) plasmids by lipofectamine 2000 (Invitrogen). At 24 h after transfection, the cells were treated with IFN-γ (10 ng/ml) for 6 h and then fixed with 4% paraformaldehyde for 10 min at room temperature and permeabilized with 0.1% Triton X-100 for 15 min. The cells were then incubated with anti-Flag tag rabbit polyclonal antibody. The cells were then incubated with goat anti-rabbit IgG (whole molecule)-tetramethyl rhodamine isocyanate antibody (T6778; Sigma). Cells were stained with 4′,6-diamidino-2-phenylindole for 15 min and examined with a Leica SP2 confocal system (Leica Microsystems).

PAM cells were treated with IFN-γ (10 ng/ml) for 1 h. The cells were mock infected or infected with ASFV for 6 h. The subsequent procedures were performed as described above.

### RNA interference (RNAi)

PAM cells were transfected with siRNA oligos using Lipofectamine RNAiMAX (Invitrogen) reagent. The RNAi sequences are as follows (only the sense strand is shown): Control, GCAGAAGAACGGCATCAAG; RNF125, CAACTGATGTAGCCAAGAG; Hes5, GGAAGCCGGTGGTGGAG.

### RT-PCR assay

PAM cells were seeded in 12-well plates (6 × 10^6^ cells/plate) for 24 h. The cells were infected with ASFV (MOI: 0.01) for 12 h before treating with IFN-γ for 1 h. 293T cells were transfected with empty vector or MGF-707-7R for 24 h. Total RNA was extracted from PAM and 293T cells using TRIzol reagent according to the manufacturer’s instruction (Sigma). Reverse transcription reactions for mRNAs were performed utilizing PrimeScript RT reagent kit (Takara). To determine relative mRNA abundance, qPCR was performed with Powerup SYBR Green Master Mix (Applied Biosystems) on an ABI StepOnePlus system according to the manufacturer's protocol. Data were analyzed with StepOnePlus software. The primer sequences were as follows: porcine *GBP1*: GAAGGGTGACAACCAGAACGAC (forward) and AGGTTCCGACTTTGCCCTGATT (reverse); porcine STAT1: TCTGGCACAGTGGCTAGAAAATC (forward) and GAAAACGGATGGTGGCAAAC (reverse); porcine *CXCL9*: TCAACACCAGCCAAAGGATG (forward) and TGACCTGTTTCTCCCACTCT (reverse); porcine *IRF1*: TCCAGCCGAGATGCTAAGTG (forward) and GTCCAAGTCCTGCCCGATGT (reverse); porcine *GAPDH*: ACATGGCCTCCAAGGAGTAAGA (forward) and GATCGAGTTGGGGCTGTGACT (reverse); human *IRF1*: GAGGAGGTGAAAGACCAGAGCA (forward) and TAGCATCTCGGCTGGACTTCGA (reverse); human *GBP1*: TAGCAGACTTCTGTTCCTACATCT (forward) and CCACTGCTGATGGCATTGACGT (reverse); human *STAT1*: ATGGCAGTCTGGCGGCTGAATT (forward) and CCAAACCAGGCTGGCACAATTG (reverse); human *CXCL9*: CTGTTCCTGCATCAGCACCAAC (forward) and TGAACTCCATTCTTCAGTGTAGCA (reverse); human *GAPDH*: GAGTCAACGGATTTGGTCGT (forward) and GACAAGCTTCCCGTTCTCAG (reverse). Human GAPDH and porcine GAPDH were used for normalization.

Afterward, the copy number of ASFV DNA was evaluated through relative quantification as previously described (51). Briefly, ASFV DNA was extracted with the QIAamp All Nucleic Acid Kit MDx Kit (Qiagen) using an automated Qiagen Universal BioRobot (Qiagen). After the DNA extraction procedure, the cartridge was processed for the RT-PCR. The target for amplification of the ASFV genome was the conserved p72 gene segment, using the following primers: 5′-ctgctcatggtatcaatcttatcga-3′ and 5′-gataccacaagatc(ag)gccgt-3′. A TaqMan probe (5′-[6-carboxy-fluorescein (FAM)]-ccacgggaggaataccaacccagtg-3′-[6-carboxy-tetramethyl-rhodamine (TAMRA)] from Applied Biosystems) was designed from an alignment of 54 available ASFV sequences for the 3′-end of p72. Analysis was performed using the MxPro software and the qPCR procedure included the following step: denaturation (95 °C), annealing (58 °C), and elongation (72 °C). The quantity of ASFV genome was calculated using the standard curve and expressed as genome copies per milliliter (/ml).

### Coimmunoprecipitation and immunoblotting analyses

For the transient transfection coimmunoprecipitation experiments, the 293T cells were transfected with the appropriate plasmid utilizing standard calcium phosphate precipitation method. Twenty-four hours after transfection, the cells were harvested and lysed in 1 ml of lysis buffer (20 mM Tris, pH 7.5, 150 mM NaCl, 1% Triton, 1 mM EDTA, 10 μg/ml aprotinin, 10 μg/ml leupeptin, and 1 mM phenylmethylsulfonyl fluoride). For each immunoprecipitation reaction, 0.4 ml of cell lysate was incubated with 0.5 μg of the indicated antibody or control IgG and 40 μl of protein G agarose beads (Santa Cruz Biotechnology, Inc) at 4 °C. After 4 h incubation, the beads were washed three times with 1 ml of lysis buffer containing 0.5 M NaCl. Samples were resolved by sodium dodecyl sulfate polyacrylamide gels (SDS PAGE) and transferred to Immobilon-P membranes (Millipore). The membranes were incubated with indicated specific primary antibodies diluted in Tris-buffered saline (TBS) supplemented with 1% milk. Membranes were washed three times with TBS and exposed 1 h to specific peroxidase-conjugated secondary antibodies. Chemiluminescence detection was performed by ECL prime (Millipore). For the endogenous coimmunoprecipitation experiments, PAM cells were infected with or without ASFV for the indicated times. The subsequent procedures were performed as described above.

### Plasmid design for traditional recombination

Plasmid pUC19 lacking its multiple cloning site was used as a backbone; the recombination cassette was inserted at the *Sal*I and *Nde*I restriction sites after the T7 promoter. The recombination cassette contains a left recombination arm that is 1000 bp upstream of ORF 7R identical to ASFV CN/GS/2018 nucleotide positions 39695 to 40694, followed by the p72 promoter identical to ASFV CN/GS/2018 nucleotide positions on the negative strand 105677 to 105465, followed by eGFP, and a SV40 termination sequence, followed by a right recombination arm that is 1000 bp downstream of 7R identical to ASFV CN/GS/2018 nucleotide positions 42270 to 43269.

### CRISPR/Cas9 transfection

gRNAs (gRNA1: GACTATGGTACTCACATACTAGG and gRNA2: GCCAATTTAAGAATTTCTTCAGG) were designed by using CRISPR RGEN Tools (http://www.rgenome.net/cas-designer/), synthesized by Sangon Biotech, and cloned into pX330 to construct targeting vectors p53-gRNA1 and p53-gRNA2. PAM was incubated with virus at 37 °C, 5% CO2 for 1 h, then the targeting vectors, and the above plasmid were transfected with Fugene HD following the manufactures protocol (available: http://www.promega.com/techserv/tools/FugeneHdTool/). A 3:1 Fugene:DNA ratio was used with 3.3 μg of DNA and 9.9 μl of Fugene HD. The complex was mixed carefully by pipetting and incubated for 10 min; 150 μl of the complex was added to the cells dropwise. Cells were then incubated at 37 °C under 5% CO2 and observed for presence of GFP. The GFP-positive single cells were picked into wells of 96-well plate, respectively. Virus particles were collected from the single cells by frozen-thaw and reseeded to PAM. After several round of pick-frozen/thaw-reseed, the PCR test cannot detect wild-type ASFV.

### Complete sequencing of ASFV genomes using next-generation sequencing

PAM cells were seeded as described and infected with ASFV, once the cytopathic effect was evident throughout the monolayer, DNA was isolated as above described from infected cells with ASFV. The extracted DNA was then used to completely sequence the viral DNA as previously described (52). In brief, the ASFV or ASFV-Δ7R DNA was sheared using enzymatic reactions assessed for the distribution of size fragmentation, and then ligation of identifying barcodes using an adapter sequence that were added to the DNA fragments. Using a Pippin Prep (Sage Science), the required size range of the library was collected and normalized. We then used this DNA library for NGS sequencing using the NextSeq (Illumina) following the manufactures protocol. Sequence analysis was performed using CLC Genomics Workbench software (CLCBio). To ensure the absence of parental CN/GS/2018 and the desired deletions in each recombinant genome, virus DNA was extracted from the virus stock and amplified by PCR with the following pair of primers: TTTGGGAAAATCCCGCGGAAAGAA and TCCTGTAGGGAGAACATTTTCTCT. The left and right homologous arm junction was amplified by PCR with the following pair of primers: TGATTGGATAGGCCAAAATCTGCC and ATGGCGGTTTATGCGAAGGATCTT; TGCTTTAAAAAACCTCCCACACCT and ACAGCATGGAGTATCAGCTTTTCA.

### Biosafety statement and facility

All experiments with live ASFVs were conducted within the enhanced biosafety level 3 (P3) facilities in the Lanzhou Veterinary Research Institute (LVRI) of the Chinese Academy of Agricultural Sciences (CAAS) approved by the Ministry of Agriculture and Rural Affairs and China National Accreditation Service for Conformity Assessment.

### Ethics statement

All animals were handled in strict accordance with good animal practice according to the Animal Ethics Procedures and Guidelines of the People's Republic of China, and the study was approved by the Animal Ethics Committee of LVRI of the CAAS.

### Virus titration

The wild-type ASFV CN/GS/2018 and MGF-505-7R-deficient viruses were quantified by using the hemadsorption (HAD) assay as described previously (53) with minor modifications. PAM cells were seeded in 96-well plates. The samples were then added to the plates and titrated in triplicate using tenfold serial dilutions. HAD was determined on day 7 postinoculation, and 50% HAD doses (HAD_50_) were calculated by using the method of Reed and Muench (54).

### Animal experiments

ASFV-Δ7R was assessed for its virulence relative to the parental ASFV CN/GS/2018 virus using 80- to 90-pound commercial breed swine. Seven pigs were inoculated intramuscularly (i.m.) with either ten HAD_50_ of ASFV-Δ7R or ASFV CN/GS/2018. Blood samples collected from three pigs that were inoculated intramuscularly (i.m.) with either ten HAD_50_ of ASFV-Δ7R or ASFV CN/GS/2018 were used for viral DNA detection and the cytokines expression detection. The body temperature and other clinical symptoms of ASFV-inoculated pigs were scored according to the clinical scoring system (32).

### ELISA

Porcine serum was collected and assayed for porcine CXCL9 and IFN-γ using Porcine CXCL9 and IFN-γ ELISA kit (Solarbio), as described by the manufacturer. ASFV p30 antibody was measured using an in-house blocking ELISA.

### Statistical analysis

The significance of differences between samples was assessed using an unpaired two-tailed Student’s *t* test. The variance was estimated by calculating the standard deviation (SD) and represented by error bars. All experiments were performed independently at least three times, with a representative experiment being shown.

## Data availability

All data have been included within the article.

## Conflict of interest

The authors declare that they have no conflict of interest with the contents of this article.
